# Evaluation of Surface Roughness of Acrylic Denture Bases Polished Using Algishine, a Novel Polishing Material: An In Vitro Study

**DOI:** 10.7759/cureus.63955

**Published:** 2024-07-06

**Authors:** Rahul Koppaka, Khushali K Shah, Nabeel Ahmed, Urvi R Echhpal

**Affiliations:** 1 Prosthodontics, Saveetha Dental College and Hospitals, Saveetha Institute of Medical and Technical Sciences, Saveetha University, Chennai, IND

**Keywords:** product development, heat-cured acrylic denture base, polishing paste, recycle, alginate

## Abstract

Introduction

Surface roughness (Ra) significantly impacts the aesthetic and functional qualities of dental prosthetics. Traditional polishing involves pumice, a material routinely used in dental practice. This study introduces Algishine as a potential cost-effective eco-friendly alternative.

Materials and methods

A 3D design software (Geomagic) created a Standard Tesselation Language (STL) file of 10 mm x 10 mm x 2 mm. 30 STL file outputs were generated. The output was milled in wax. This was then flasked and processed. 30 acrylic resin specimens were fabricated and divided into two groups. Group A was polished using traditional pumice, and Group B was polished using Algishine. The Ra of each sample was measured using surface profilometry, with three readings per sample averaged for each group.

Results

Kruskal-Wallis test was performed to compare the two groups with the pre-testing samples, which showed p<0.05; indicating that there was a significant difference between the two groups. The average Ra value for unpolished acrylic was 7.105, while the specimens polished with pumice showed an Ra value of 2.218; specimens polished with novel material Algishine showed an Ra value of 1.743. This illustrates that Algishine achieves surface smoothness significantly superior to commonly used polishing agent and pumice.

Discussion

The results of our study demonstrate that Algishine, a novel polishing material derived from recycled alginate, effectively reduces the Ra of acrylic resin. This finding has significant implications, both clinically and environmentally. The primary clinical benefit of a smoother acrylic resin surface is the enhanced aesthetic appearance and increased patient comfort. A polished surface reduces plaque accumulation, thereby decreasing the risk of oral infections and improving the longevity of the dental prosthesis. The results show that Algishine achieves surface smoothness comparable to or better than pumice indicating that it can maintain, if not enhance, these clinical outcomes. Dental professionals can confidently use Algishine, knowing it meets the high standards required for patient care.

Conclusion

Algishine effectively reduces the Ra of acrylic resin, suggesting it is a viable, eco-friendly alternative to traditional pumice for dental polishing procedures. This indicates potential benefits in maintaining clinical outcomes while promoting environmental sustainability.

## Introduction

Various materials have been used for denture bases, but poly(methyl methacrylate) (PMMA) remains the most common choice [1]. This is because it is easy to manipulate, repair, and polish, and it is both aesthetic and stable. However, it has been found to have drawbacks such as polymerization shrinkage, allergic reactions to residual monomer, and poor wear resistance [2]. However, the quality of the final product greatly depends on the surface finish achieved during the polishing process, which relies heavily on the skill of the technician [3]. In clinical dentistry, there are many situations where it is necessary to adjust denture base acrylic resin. This adjustment process changes the originally finished and polished surface of the denture base, resulting in a rougher texture [4]. This roughened surface can lead to plaque buildup and staining [5].

The surface finish of acrylic resin prosthetics is vital for achieving a smooth, glossy appearance that enhances the visual appeal, making dental prosthetics look more natural [6]. This polished surface minimizes irritation to oral tissues, improving overall patient comfort. Additionally, a well-finished surface is less prone to plaque accumulation and bacterial colonization, which are crucial for maintaining oral hygiene and preventing infections [7].

Traditionally, pumice has been the preferred material for polishing acrylic resins [8]. This fine powder, derived from volcanic ash, is effective in smoothing rough surfaces of acrylic resin, resulting in a polished finish. However, pumice use has several drawbacks. The extraction and processing of pumice have ecological impacts, such as habitat disruption and carbon emissions from mining activities. Additionally, as a finite natural resource, the continuous extraction of pumice raises sustainability concerns.

Given the environmental concerns associated with pumice, there is increasing interest in finding sustainable alternatives [9]. This study introduces Algishine, a novel polishing agent made from recycled pulverized alginate impressions [10]. Alginate, derived from seaweed, is commonly used in dental practices to create impressions of teeth and the oral cavity. Typically, these alginate impressions become waste after use. Algishine repurposes this waste into a polishing material, addressing both environmental sustainability and resource efficiency.

This study aimed to evaluate the efficiency of Algishine against pumice in reducing the surface roughness (Ra) of acrylic resin. The null hypothesis stated that there was no significant difference in the polishing ability of Algishine and pumice.

## Materials and methods

Sample size calculation

**Figure 1:**
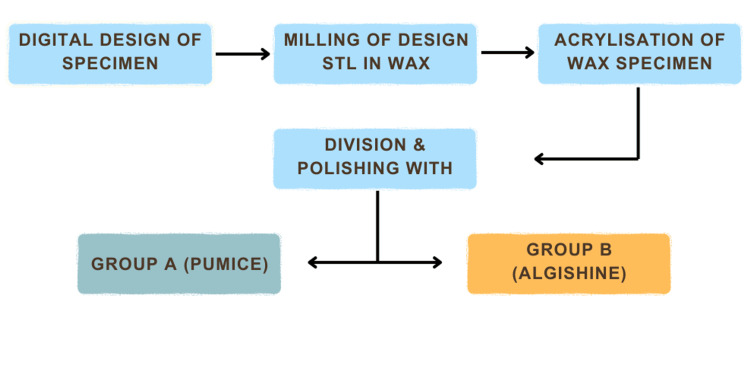
Methodology Figure credit: Authors' creation

This study employed an in vitro experimental design. The sample size was determined using the means comparison formula with Statistical Software IBM® SPSS® version 25.0 (IBM, USA), setting an alpha level of 0.05 and a test power of 0.80. Consequently, a sample size of 30 was obtained, then divided into three groups of 10 each (Figure 1).

Fabrication of Algishine

**Figure 2:**
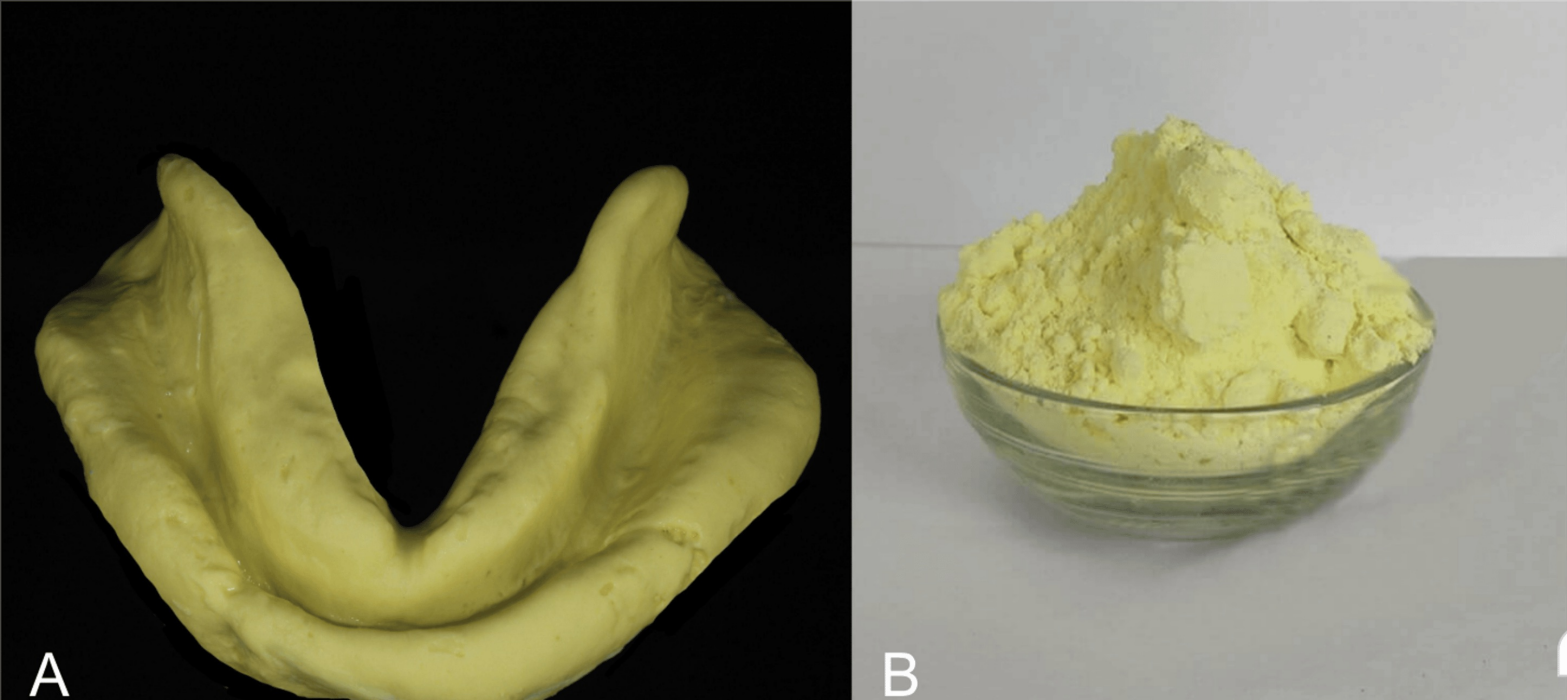
A: Alginate impression; B: Algishine powder

Alginate impressions were disinfected using 2% glutaraldehyde for 30 minutes (Figure 2A). Disinfected alginate impressions were retrieved and pulverized to a fine powder. The pulverizing device (Foshan Xingle Machinery XL-88) includes a sealable chamber and a freely rotating blade within that chamber. The impression was then first sectioned using a 10 no. Bard Parker blade. Then the alginate impressions were pulverized for 180 seconds using this device to pass through a sieve (Labotec sieving mesh, SABS 197-1971) to achieve a 15µm particle size. A dehydrated powder is produced using this method (Figure 2B).

Fabrication of the acrylic specimen

**Figure 3:**
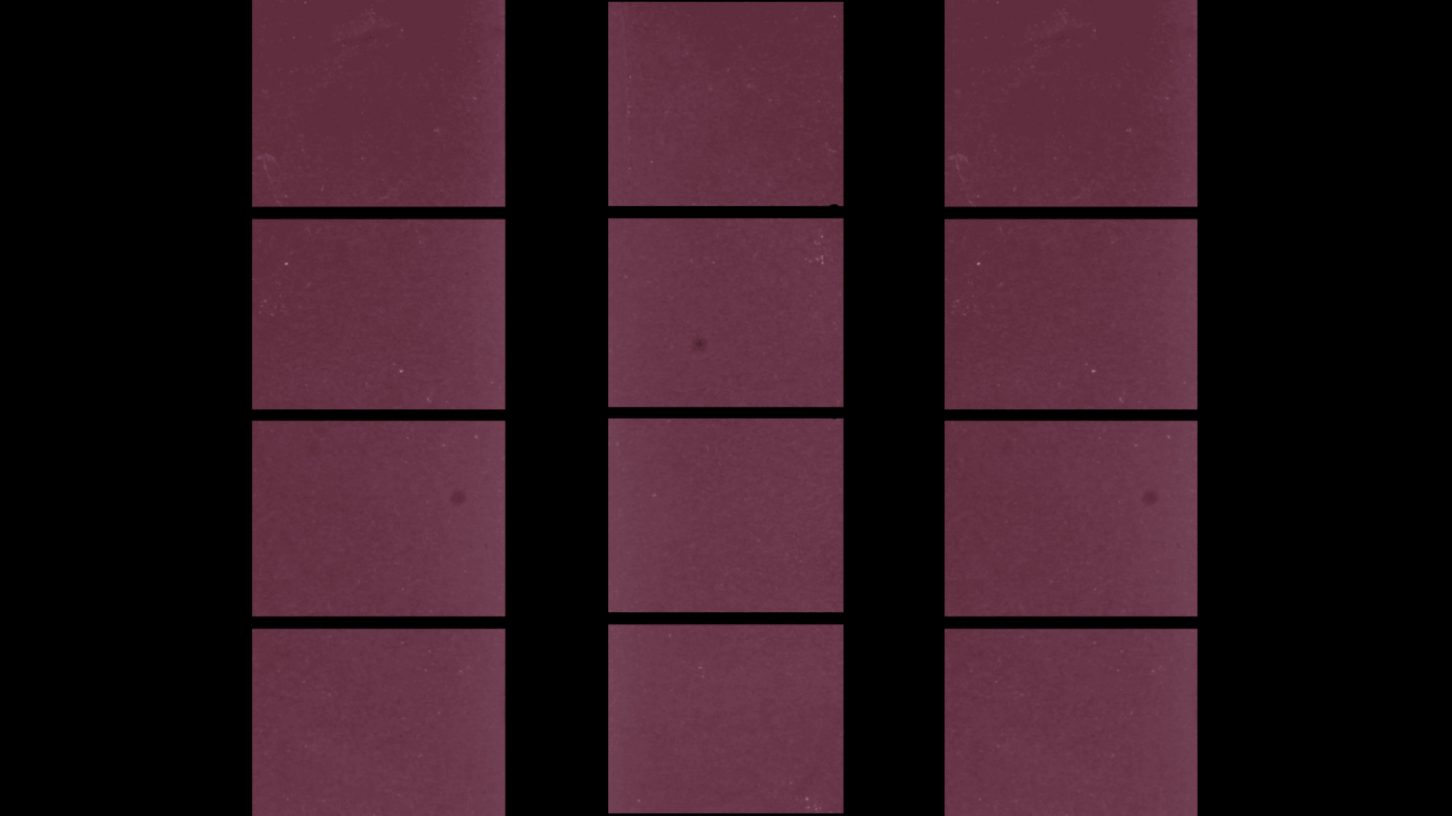
Acrylic specimens 10 mm x 10 mm x 2 mm

A 3D design software (Geomagic) was used to create a Standard Tesselation Language (STL) file of 10 mm x 10 mm x 2 mm. 30 STL file outputs were generated. The output was milled in wax using the iMes-iCore 350 milling machine. The wax patterns generated were then flasked and processed using heat-cured acrylic material (Dental Products of India (DPI) heat-cure acrylic). 30 acrylic resin specimens were fabricated. These were then assigned to each group, Group A (pumice), Group B (Algishine), and Group C (pre-polishing); with 10 in each group (Figure 3).

Polishing protocol

**Figure 4:**
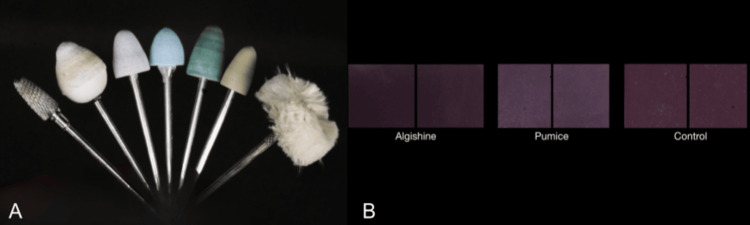
A: Polishing burs; B: Changes in surface texture and translucency in polished samples

Samples were polished with sandpaper of various grits, 100→400→1,000. Shofu Acrylic Polishing bur kit was used with four burs, extra coarse (grey), coarse (blue), fine (dark green), and extra fine (light green) (Figure 4A). Group A was then polished with Pumice and Group B with Algishine. The samples were then polished with a buff (wet buffing was followed by dry buffing). 

In Group A (Pumice), the 10 samples were finally polished with pumice using standard protocol. In Group B (Algishine), the 10 samples were finally polished with Algishine using an identical protocol. In Group C (pre-polishing), no Polishing was done.

Change in surface texture and translucency was noted for samples in all three groups, with samples polished with Algishine showing fewer craze lines and an overall smoother finish (Figure 4B).

Analysis of Surface Roughness

**Figure 5:**
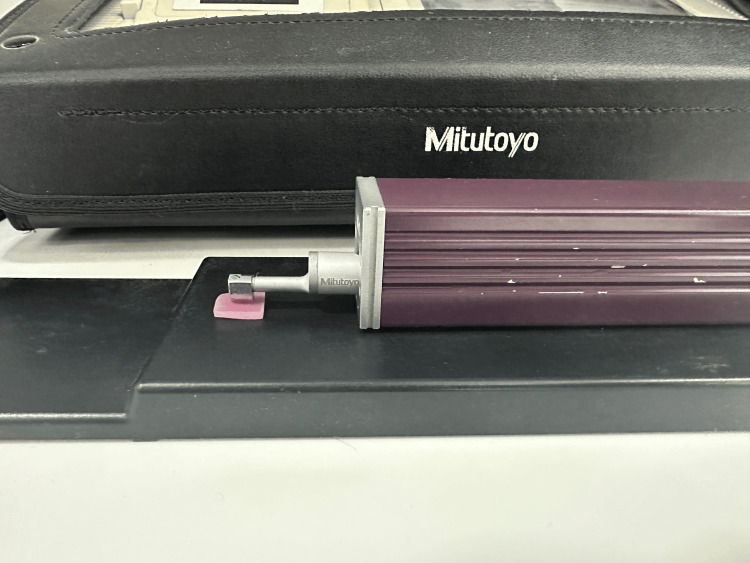
Surface profilometer

A profilometer (Mitutoyo- SURFTEST SJ-210 ) is used to measure the Ra of the polished samples, providing quantitative data on the efficacy of each polishing agent. The Ra of each sample was measured using the profilometer at three different points per sample, averaging the readings for each group. The profilometer features a vertical measurement range of ±300 µm, a tip radius of 5 µm, and a cone angle of 90° (Figure 5).

Profilometric profiles were acquired over a traversing length of 10 mm. With a cut-off length of 0.8 mm, the arithmetic mean Ra values were determined. Each specimen underwent three measurements, with an incremental distance of 1 mm between each reading. The mean roughness value was calculated as the average of these three measurements. Measurements were taken in a direction perpendicular to the polishing direction.

Statistical analysis

The Ra values obtained from the profilometer readings were analyzed using descriptive and inferential statistics with IBM® SPSS® version 25.0 (IBM, USA), considering a significance level of p < 0.05. The Ra of the resins was represented by the mean and standard deviation. The comparison of the average Ra values of the resin types subjected to a specific type of polishing with their respective controls was conducted using a non-parametric test. The Ra values were compared between different polishing methods using the Kruskal-Wallis test for independent samples.

## Results

Surface roughness

**Figure 6:**
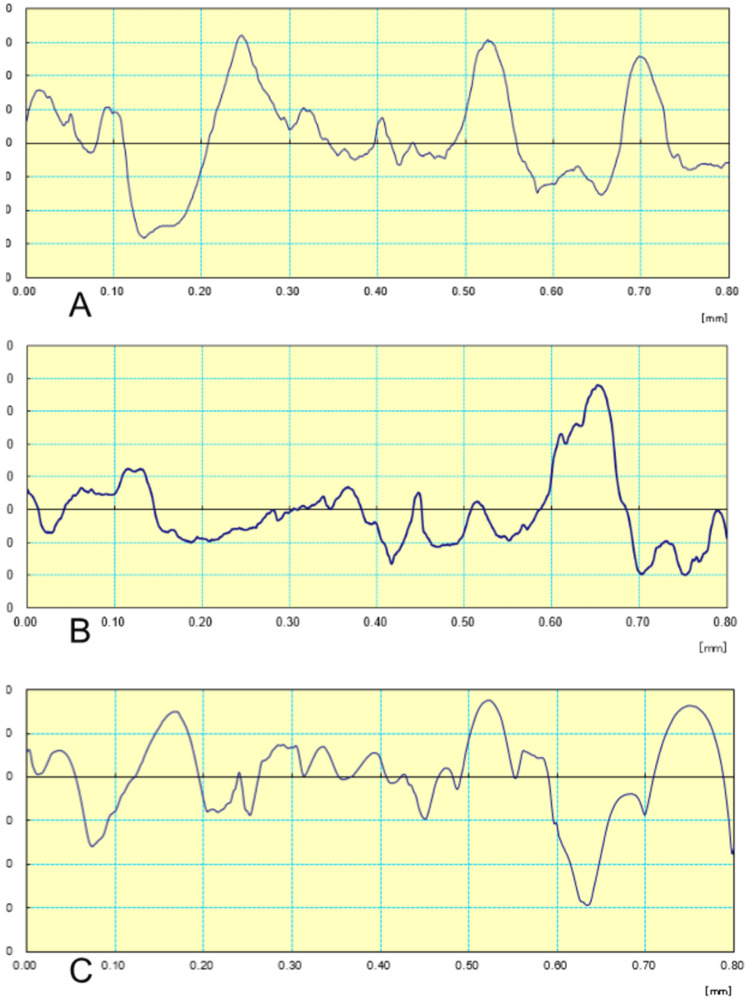
Ra readings of Groups A, B, and C using a profilometer Ra: Surface roughness

Profilometric profiles were measured across a 10 mm span. Using a cut-off length of 0.8 mm, the arithmetic mean Ra values were calculated. The following waveform graph denotes the Ra values generated during the testing of the three groups (Figure 6). The Kruskal-Wallis test was performed to compare the means of the three groups, which showed p value < 0.05; indicating a significant difference.

**Figure 7:**
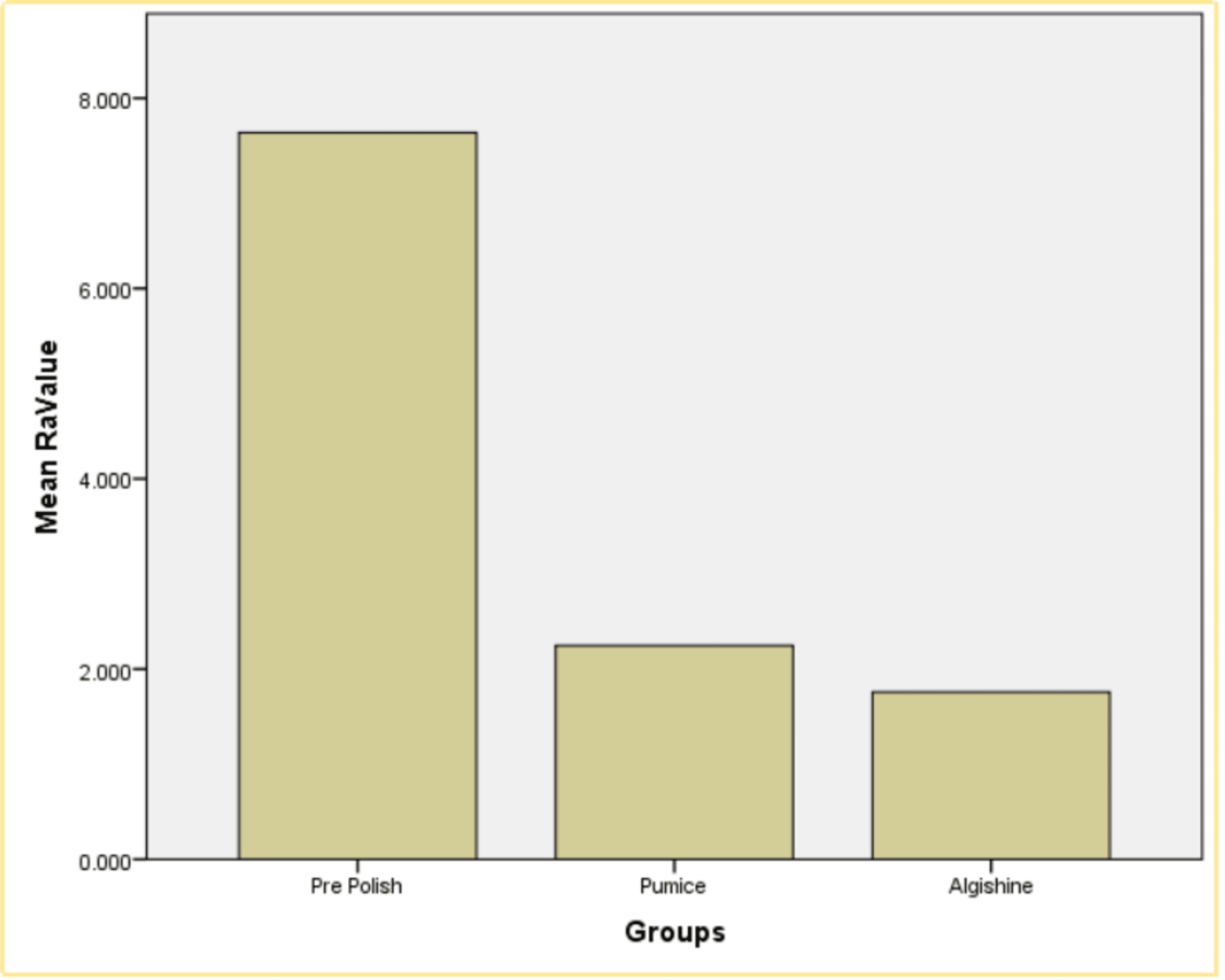
Graphical representation of the comparison between the three groups

The table provides Ra data for acrylic resin samples polished with different agents. Group A, using pumice, has an average Ra value of 2.218 with a standard deviation of 0.344, indicating moderate surface smoothness and variability. Group B, using Algishine, shows the best results with an average Ra value of 1.743 and a low standard deviation of 0.205, indicating the smoothest and most consistent surface finish. Group C, representing the pre-polished state, has the highest average Ra value of 7.105 and a standard deviation of 0.60443, indicating significant initial roughness and high variability. Overall, Algishine outperforms pumice in achieving a smoother and more consistent surface, while the pre-polished samples exhibit the roughest texture (Table 1). The comparative data suggest that Algishine achieves a significantly smoother, surface finish compared to pumice (Figure 7).

**Table 1:** Mean Ra values and SD Ra: Surface roughness

Group	Average Ra Value	SD
A (pumice)	2.218	0.344
B (Algishine)	1.743	0.205
C (pre-polishing)	7.105	0.60443

Potential implications

If Algishine demonstrates comparable or superior performance to pumice in reducing Ra, it could revolutionize dental polishing practices by, promoting a sustainable method and recycling alginate waste, Algishine supports environmental sustainability and reduces the ecological footprint of dental practices. It also has economic benefits by utilizing recycled materials could potentially lower costs for dental laboratories and practices.

## Discussion

The results of our study demonstrate that Algishine, a novel polishing material derived from recycled alginate, effectively reduces the Ra of acrylic resin. This finding has significant implications, both clinically and environmentally.

The primary clinical benefit of a smoother acrylic resin surface is the enhanced aesthetic appearance and increased patient comfort [11]. A polished surface reduces plaque accumulation, thereby decreasing the risk of oral infections and improving the longevity of the dental prosthesis [12]. Other methods have also been tested to find natural alternatives to pumice as a polishing material. Polishing paste made from alginate powder has shown positive results when compared to traditional methods [13]. This topic has received limited attention in dentistry, and there is a lack of literature evaluating Ra with the experimental paste. Consequently, it is anticipated that research in this area will persist in testing new experimental compounds, given the significant ongoing efforts in this field [14].

The results show that Algishine achieves surface smoothness comparable to or better than pumice indicating that it can maintain, if not enhance, these clinical outcomes. Dental professionals can confidently use Algishine, knowing it meets the high standards required for patient care. One of the most compelling aspects of Algishine is its origin from recycled alginate impressions. Alginate, commonly used in dental impressions, typically ends up as waste after a single use. By recycling this material into a polishing agent, Algishine contributes to waste reduction and promotes a circular economy within the dental industry. This sustainability aspect is increasingly important as dental practices seek to reduce their environmental footprint. Algishine not only addresses the environmental impact associated with alginate disposal but also reduces the need for mining and processing pumice, further lessening ecological strain.

Recycling alginate impressions into a polishing material could potentially lead to cost savings for dental practices. Traditional pumice, while relatively inexpensive, still involves costs related to mining, processing, and transportation. By reusing alginate waste generated in-house, dental laboratories and practices can reduce material costs. Additionally, adopting Algishine could lower waste management expenses due to reduced disposal needs.

Maintaining high-quality dental prosthetics is crucial for patient satisfaction and clinical success [15]. The smooth surface achieved with Algishine enhances the durability and wear resistance of acrylic resin prosthetics. A smoother surface is less prone to scratches and microbial colonization, thereby extending the functional life of the prosthesis [16]. This improved quality directly translates to fewer adjustments and repairs, saving time and resources for both dental professionals and patients [17].

The main limitation of this study is the lack of control over the force exerted during the acrylic polishing process, as well as the absence of a device to measure this force. To mitigate this, specific criteria were implemented: a single operator performed all polishing tasks, and short breaks were taken after every five acrylic samples to prevent operator fatigue. Another limitation was that only the microroughness of heat-curing PMMA was evaluated. Nonetheless, the use of Algishine aligns with broader trends in healthcare towards sustainable practices and materials. As the dental industry becomes more conscious of its environmental impact, adopting eco-friendly materials like Algishine can position dental practices as leaders in sustainability. This can enhance the practice's reputation among environmentally conscious patients and potentially attract a broader clientele. We need to ensure that the new material does not compromise the quality of the prosthetics ensuring continued patient satisfaction and clinical success.

## Conclusions

In summary, Algishine offers a promising sustainable alternative to traditional pumice for polishing acrylic resin in dental applications. It offers comparable, if not superior, surface quality, contributing to dental prosthetics' aesthetic and functional excellence. Additionally, its environmental and economic benefits make it an attractive option for dental practices committed to sustainability. Further research will help solidify Algishine’s place in the dental industry, ensuring it meets the long-term needs of both patients and practitioners.

While this study establishes the immediate effectiveness of Algishine in polishing acrylic resin, further research is necessary to understand its long-term implications fully. Key areas for future investigation include examining how the use of Algishine affects the long-term durability and wear resistance of acrylic resin prosthetics compared to those polished with pumice and conducting clinical trials to assess patient feedback on the comfort and appearance of prosthetics polished with Algishine over extended periods.
